# RNA Modifications as Molecular Regulators of Alveolar Epithelial Injury and Aberrant Repair in Pulmonary Fibrosis

**DOI:** 10.3390/biom16081176

**Published:** 2026-08-12

**Authors:** Qi Huang, Shuguang Wang, Yuman Huang, Shibo Xiao, Ruohan Xia, Xianwang Wang

**Affiliations:** Health Science Center, Yangtze University, Jingzhou 434023, China; 2025711039@yangtzeu.edu.cn (Q.H.); sg.wang.stu@yangtzeu.edu.cn (S.W.); 2025711040@yangtzeu.edu.cn (Y.H.); 2025711049@yangtzeu.edu.cn (S.X.)

**Keywords:** RNA modifications, pulmonary fibrosis, alveolar epithelial cells, AT2 cells, aberrant repair, m6A, epitranscriptomics, epithelial–mesenchymal communication

## Abstract

Pulmonary fibrosis is a progressive interstitial lung disease characterized by persistent alveolar epithelial injury, aberrant repair, and excessive extracellular matrix deposition. Increasing evidence indicates that disease progression is closely associated with alveolar type II (AT2) cell dysfunction, impaired AT2-to-AT1 differentiation, and the persistence of transitional epithelial populations, including KRT8^+^ intermediate populations. Because the formation and resolution of these transitional epithelial populations require dynamic regulation of stress-responsive transcripts and differentiation-associated RNA programs, they provide a biologically relevant context for investigating RNA modification-mediated post-transcriptional regulation. RNA modifications have emerged as post-transcriptional regulatory layers that modulate RNA stability, processing, translation efficiency, and stress-response gene expression, thereby influencing epithelial stress adaptation and repair-related state transitions. Among these modifications, N6-methyladenosine (m6A) is the best-characterized layer, with evidence linking it to epithelial injury responses, senescence-associated transcript remodeling, and differentiation impairment. In contrast, non-m6A modifications, including m5C, m1A, m7G, pseudouridine (Ψ), and A-to-I RNA editing, remain emerging regulatory layers with limited AT2 cell-specific functional validation. This review summarizes current evidence connecting RNA modifications with alveolar epithelial injury, transitional-state persistence, epithelial–mesenchymal communication, and fibrotic remodeling. Rather than interpreting RNA modifications as isolated pathogenic drivers, we highlight their context-dependent roles in RNA fate control, epithelial stress adaptation, and aberrant repair in pulmonary fibrosis.

## 1. Introduction

Pulmonary fibrosis (PF) is a progressive interstitial lung disease characterized by alveolar architectural destruction and excessive extracellular matrix (ECM) deposition, closely associated with dysregulated alveolar epithelial injury–repair processes and progressive decline in lung function [1]. Idiopathic pulmonary fibrosis (IPF), the most well-characterized clinical subtype, shows increasing incidence and prevalence worldwide, partly due to population aging and improved detection using high-resolution imaging techniques [1,2]. Current anti-fibrotic and emerging targeted therapies, including the PDE4B inhibitor nerandomilast, have shown disease-modifying potential mainly by slowing functional decline rather than reversing established fibrosis [3,4]. Additional clinical evidence further supports that current therapeutic strategies mainly attenuate disease progression but remain insufficient to restore early epithelial injury responses and regenerative repair [5]. Collectively, these findings highlight the need to clarify molecular regulatory mechanisms underlying epithelial repair failure in pulmonary fibrosis.

Historically, pulmonary fibrosis has often been interpreted through fibroblast and myofibroblast activation [6,7]. However, increasing evidence indicates that aberrant alveolar epithelial repair is not merely a secondary consequence of mesenchymal activation but represents an important upstream and an important component of fibrotic remodeling [8]. Under physiological conditions, alveolar type II (AT2) cells function as progenitor-like epithelial cells that proliferate and differentiate into alveolar type I (AT1) cells following injury, thereby maintaining alveolar structural integrity [9]. However, under persistent injury or a pro-fibrotic microenvironment, this regenerative process may become impaired, leading to aberrant transitional epithelial populations and reduced regenerative capacity of the alveolar epithelium [10]. Among these, KRT8^+^ transitional epithelial cells have been observed in fibrotic lung tissues, and temporo-spatial profiling of IPF regenerating alveolar niches supports the existence of aberrant epithelial intermediates within fibrotic regions [10,11]. These transitional epithelial populations are typically transient during normal repair but may persist under pathological conditions [10]. These aberrant epithelial alterations not only reflect impaired repair processes but may also contribute to local inflammatory regulation and altered epithelial–mesenchymal communication [8,11].

RNA modifications, as key components of the epitranscriptomic landscape, regulate RNA stability, splicing, RNA processing, and translational efficiency, thereby modulating cellular stress responses and epithelial transitions [12,13]. Current evidence suggests that m6A modification may participate in alveolar epithelial stress responses and differentiation impairment; however, many findings are derived from non-AT2-specific models or correlative studies [14,15]. Importantly, alveolar epithelial cells are not considered a major direct source of myofibroblasts through canonical epithelial–mesenchymal transition (EMT); instead, they may exhibit partial EMT-like transcriptional programs and altered secretory phenotypes [16,17]. In contrast, non-m6A RNA modifications, including m5C, m1A, m7G, pseudouridine (Ψ), and A-to-I RNA editing, remain at an early stage of investigation in pulmonary fibrosis, with current evidence largely limited to expression-based or correlative studies and lacking functional causality [18,19]. Moreover, single-cell epitranscriptomic approaches remain technically immature for most RNA modifications, limiting cell-state-resolved interpretation in alveolar epithelial systems [18,19].

Previous reviews have summarized the roles of RNA modifications or broader epigenetic mechanisms in pulmonary fibrosis, with a primary focus on individual RNA modification types, regulatory enzymes, or their associations with fibrotic signaling pathways. However, how RNA modifications contribute to the dynamic regulation of alveolar epithelial injury adaptation and aberrant repair remains incompletely characterized, particularly regarding their potential roles in regulating AT2 cell responses, epithelial plasticity, and repair-associated transitions. In this review, we focus on RNA modifications as potential regulators of alveolar epithelial injury adaptation and aberrant repair, emphasizing their potential involvement in AT2 cell stress responses, epithelial plasticity, KRT8^+^ transitional epithelial populations, impaired AT2-to-AT1 differentiation, and epithelial–mesenchymal communication.

By integrating epitranscriptomic regulation with alveolar epithelial repair biology, we aim to summarize current evidence regarding how RNA modifications may influence epithelial responses and fibrotic remodeling through regulation of RNA fate and stress-response programs.

**Figure 1:**
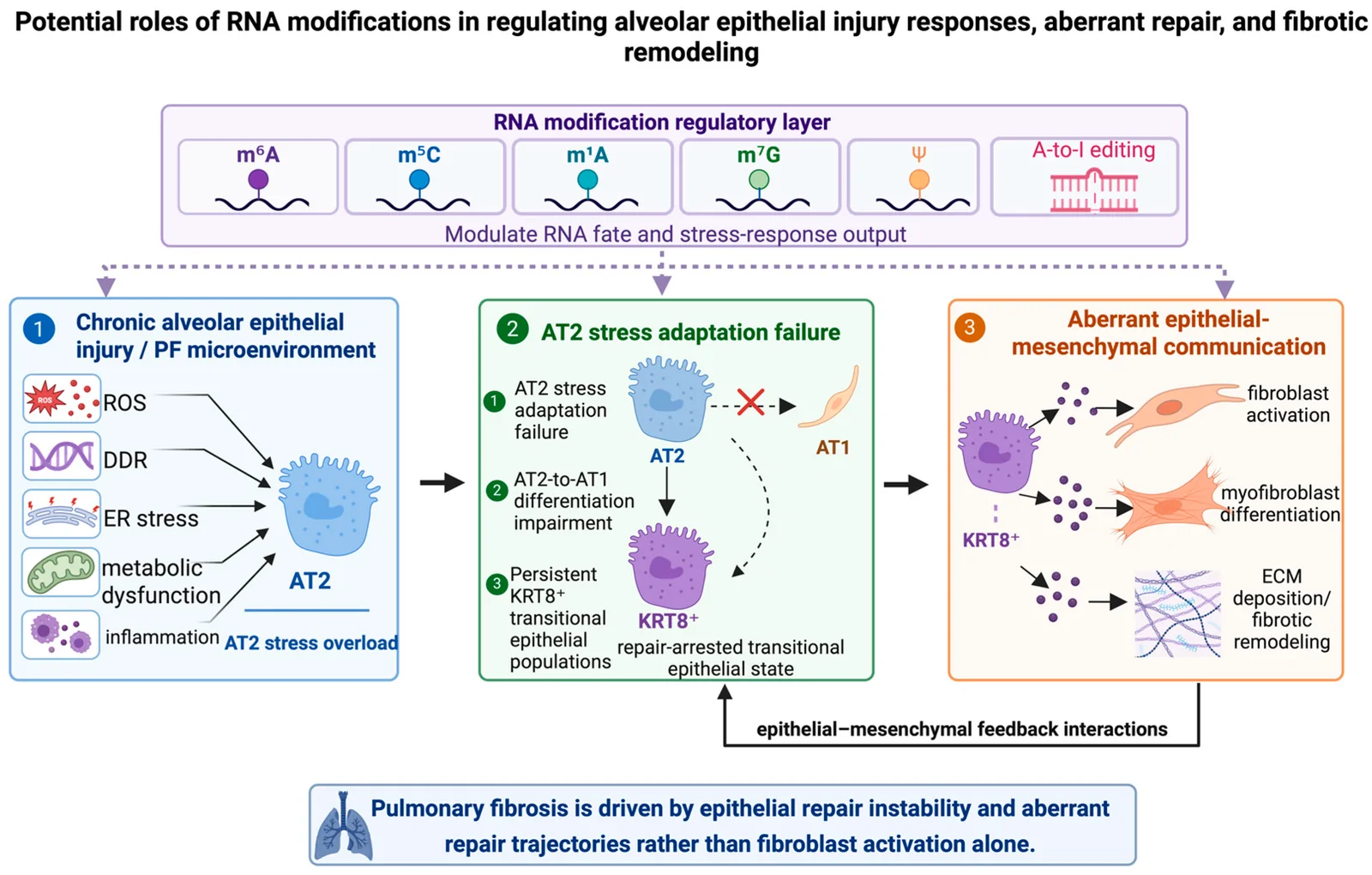
Potential roles of RNA modifications in regulating alveolar epithelial injury responses, aberrant repair, and fibrotic remodeling. Persistent epithelial injury, including oxidative stress, endoplasmic reticulum stress, DNA damage responses, and ferroptotic stress, disrupts alveolar epithelial homeostasis. In this context, RNA modification regulatory layers, including m6A, m5C, m1A, m7G, pseudouridine (Ψ), and A-to-I RNA editing, may modulate RNA fate through effects on RNA stability, splicing and RNA processing, translation efficiency, immune sensing, and stress-response transcript regulation. These post-transcriptional regulatory events may contribute to altered AT2 stress adaptation, impaired AT2-to-AT1 differentiation, and persistence of KRT8^+^ transitional epithelial states. At the tissue level, these alterations may contribute to aberrant epithelial–mesenchymal crosstalk, fibroblast activation, and extracellular matrix deposition, thereby promoting fibrotic remodeling in pulmonary fibrosis. Solid arrows indicate sequential biological processes or regulatory relationships, whereas dashed arrows represent potential or indirect associations. Different colors highlight distinct biological modules, including epithelial injury/stress responses (blue), AT2 stress adaptation failure and transitional epithelial programs (green), and epithelial-mesenchymal communication and fibrotic remodeling (orange).

Based on this context, this review summarizes current evidence regarding RNA modifications as potential regulatory layers in AT2 cell injury responses, aberrant epithelial repair, epithelial–mesenchymal communication, and fibrotic signaling regulation, while highlighting key gaps in cell-type specificity, site-level mapping, and functional validation (Figure 1).

## 2. AT2 Cell Injury and Failure of Stress Adaptation During Pulmonary Fibrosis Progression

Alveolar type II (AT2) epithelial cells contribute to alveolar structural homeostasis and epithelial regeneration following injury through self-renewal and differentiation into alveolar type I (AT1) cells. AT2-related epithelial diversity and heterogeneity are further supported by large-scale human lung cell atlas studies [1,2]. Recent single-cell lineage tracing studies indicate that AT2 cells do not undergo a single discrete injury event during lung repair; instead, they experience continuous microenvironmental stress leading to epithelial remodeling and functional dysregulation, ultimately resulting in impaired regenerative trajectories [3,4].

### 2.1. Stress Network-Mediated Mechanisms of AT2 Injury

AT2 cell injury is associated with multiple interacting stress responses, including energy metabolic dysregulation related to oxidative stress and mitochondrial dysfunction [5,6], cell-cycle arrest associated with the DNA damage response [7,8], and proteostasis imbalance caused by endoplasmic reticulum stress and metabolic reprogramming [9,10]. Together, these stress pathways may interact to amplify cellular injury responses and reduce the adaptive capacity of AT2 cells to microenvironmental perturbations [11]. Increasing evidence suggests that RNA modifications may participate in these stress responses by regulating the stability, processing, and translation of stress-associated transcripts.

(1) Oxidative stress and mitochondrial dysfunction mechanisms

Oxidative stress is characterized by the accumulation of reactive oxygen species (ROS) and impaired mitochondrial function, leading to reduced ATP production and disruption of cellular energy metabolic homeostasis [5,6]. This process reduces the survival and reparative capacity of AT2 cells within the injured microenvironment.

(2) DNA damage response-driven proliferative inhibition mechanisms

The DNA damage response (DDR) induces cell cycle arrest through the p53/p21 and p16/Rb pathways, thereby suppressing the proliferative and regenerative capacity of AT2 cells [7,12]. This state limits the potential of AT2 cells to re-establish a normal differentiation trajectory, particularly when inflammatory cytokine signaling overlaps with the repair phase [13].

(3) Endoplasmic reticulum stress and metabolic imbalance mechanisms

Endoplasmic reticulum stress is activated through the unfolded protein response (UPR) and is accompanied by metabolic reprogramming, together leading to proteostasis disruption [9,10]. This process exhibits substantial crosstalk with oxidative stress and the DNA damage response, forming a cross-pathway stress amplification effect [14,15].

### 2.2. Integrated Stress Responses and AT2 Repair Dysfunction

These stress responses do not act independently; instead, they interact within the alveolar microenvironment and collectively influence AT2 repair capacity. Based on current evidence, AT2 cell injury can be viewed as a progressive process in which persistent stress responses may impair AT2 stress adaptation and contribute to epithelial repair instability. This process emphasizes that AT2 cell injury is not a single molecular event, but rather reflects the combined effects of multiple interacting stress responses [9,11].

### 2.3. Summary of AT2 Injury-Associated Repair Dysfunction

AT2 cell injury represents an early disruption of alveolar epithelial regenerative processes, reflecting the combined effects of multiple stress factors, including oxidative stress, DNA damage, and endoplasmic reticulum–metabolic dysregulation [9,15]. These injury-associated stress responses may contribute to subsequent epithelial dysfunction and abnormal repair processes during pulmonary fibrosis, which may subsequently influence transitional epithelial states and epithelial–mesenchymal communication during disease progression [8,17].

## 3. Role of RNA Modifications in Alveolar Epithelial State Regulation

RNA modifications, as an important component of the epitranscriptome, regulate cellular stress responses and repair-associated responses by modulating RNA stability, splicing, nuclear export, and translational efficiency [16,17]. Beyond individual RNA fate control events, coordinated epitranscriptomic regulation may contribute to cellular adaptability under stress conditions by integrating multiple RNA regulatory processes.

Recent studies have suggested that RNA modifications do not act solely at the level of individual gene expression but may regulate broader post-transcriptional networks, thereby influencing cellular adaptation and maintenance of cellular plasticity [17,18]. In the context of alveolar epithelial injury, coordinated RNA regulation may help cells dynamically adjust RNA output in response to environmental stress, rather than acting as a deterministic switch controlling cell fate.

In alveolar epithelial cells, RNA modifications may function as context-dependent regulators of transcriptional output under stress conditions by modulating RNA stability, processing, and translation, rather than directly determining cell fate [16,18].

### 3.1. m6A Modification: The Core Post-Transcriptional Regulatory Layer

N6-methyladenosine (m6A) is the most extensively studied RNA modification, whose dynamic regulation depends on the METTL3/METTL14 writer complex, the FTO/ALKBH5 demethylases, and the YTHDF family of reader proteins [19,20].

In pulmonary fibrosis-related studies, m6A has been associated with regulation of the stability and translational efficiency of stress-related mRNAs, potentially influencing AT2 cellular responses and repair capacity following injury [21,22].

Existing studies have reported dysregulation of m6A writers, including METTL3 and METTL14, in lung injury or fibrosis-related models, with potential associations with epithelial senescence and impaired repair [19,23].

However, most evidence is derived from models not specific to AT2 cells; therefore, its causal role still requires further validation, particularly because bulk m6A profiling may mask cell-to-cell methylation heterogeneity that becomes detectable only at single-cell resolution [24].

In this context, m6A may function as a context-dependent regulator of RNA stability and stress-response programs rather than a deterministic switch controlling epithelial cell fate [20,23].

### 3.2. Non-m6A RNA Modifications: Auxiliary Regulatory Layer

In addition to m6A, modifications such as m5C, m1A, m7G, pseudouridine (Ψ), and A-to-I RNA editing are increasingly considered to participate in alveolar epithelial injury processes; however, the overall evidence remains mainly correlative [25,26].

#### 3.2.1. m5C Modification

m5C is primarily regulated by the NSUN family of methyltransferases and reader proteins such as YBX1, and it can influence RNA stability and nuclear export processes [27].

Current studies suggest that it may be involved in stress- and inflammation-related transcriptional regulation; however, direct mechanistic evidence in pulmonary fibrosis remains insufficient [27].

#### 3.2.2. m1A Modification

m1A is mainly enriched in tRNAs and specific mRNA structural regions, and it can regulate translation initiation efficiency and protein synthesis rate [28].

In lung tissue, it may be associated with mitochondrial metabolism and energy homeostasis; however, AT2 cell-specific mechanistic studies are still lacking [28].

#### 3.2.3. m7G and Ψ Modifications

m7G and pseudouridine (Ψ) mainly affect RNA structural stability and translational efficiency and may participate in the global post-transcriptional regulatory network under stress conditions [29].

However, their functions in pulmonary fibrosis remain at an early exploratory stage, and direct mechanistic evidence remains limited [29].

### 3.3. Summary

Overall, current evidence supports RNA modifications as regulatory mechanisms that influence cellular stress adaptation and epithelial repair responses. Their primary function may involve regulating cellular adaptation under stress conditions rather than acting as a single determinant of cell fate decisions [28,30] (Table 1).

## 4. RNA Modification-Associated Regulation of AT2 Transition During Aberrant Repair

Alveolar epithelial regeneration requires coordinated activation, proliferation, and differentiation of alveolar type II (AT2) cells following injury. Under physiological conditions, AT2 cells function as facultative progenitor cells and undergo dynamic transitions involving activation, proliferation, and differentiation toward alveolar type I (AT1) cells, thereby contributing to restoration of epithelial integrity and maintenance of alveolar function. Lineage-tracing and single-cell transcriptomic studies have demonstrated that AT2-derived transitional epithelial populations represent intermediate stages during AT1 regeneration, highlighting the importance of controlled epithelial plasticity in maintaining alveolar homeostasis [3,4].

In pulmonary fibrosis, this regenerative trajectory becomes dysregulated. Persistent epithelial stress, inflammatory stimulation, and altered tissue microenvironments may interfere with the progression from activated AT2 cells toward mature AT1 cells, resulting in delayed or incomplete epithelial maturation. Recent studies have demonstrated that regulation of differentiation-associated transcriptional networks is essential for effective epithelial repair. For example, thyroid hormone receptor β-mediated activation of KLF2 and CEBPA promotes AT2-to-AT1 differentiation and enhances alveolar regeneration, suggesting that impaired regulation of epithelial differentiation programs may contribute to defective repair in fibrotic conditions [37].

Beyond transcriptional regulation, post-transcriptional mechanisms have increasingly been recognized as important regulators of epithelial responses during injury and repair. RNA modifications may influence epithelial adaptation by regulating RNA stability, translation efficiency, and stress-responsive gene expression. Through these mechanisms, epitranscriptomic regulation may contribute to AT2 cell adaptation to injury-associated environmental changes, although whether specific RNA modifications directly control epithelial lineage transitions remains incompletely understood.

### 4.1. AT2-to-AT1 Differentiation as a Regenerative Trajectory

AT2 cells represent a critical regenerative population within the alveolar epithelium. Following injury, activated AT2 cells undergo proliferation and phenotypic transitions that enable subsequent differentiation toward AT1 cells. This process requires coordinated regulation of developmental programs, transcriptional networks, and environmental signals to ensure restoration of the alveolar barrier. Studies using lineage tracing and single-cell approaches have identified transitional epithelial populations as intermediate stages during AT2-to-AT1 conversion, supporting the concept that alveolar repair occurs through progressive cellular transitions rather than direct lineage replacement [3,4].

During fibrotic remodeling, the transition from activated AT2 cells toward mature AT1 cells may become delayed or incomplete. Such impairment reflects failure to resolve injury-associated epithelial programs rather than irreversible determination of a specific pathogenic cell fate. Experimental evidence indicates that modulation of differentiation-related regulatory networks can influence epithelial recovery. In particular, activation of the thyroid hormone receptor β-KLF2/CEBPA axis promotes AT2-to-AT1 differentiation and improves epithelial regeneration, emphasizing the importance of maintaining appropriate differentiation trajectories during repair [37].

RNA modifications may represent an additional regulatory layer affecting AT2-associated epithelial responses. By regulating transcript stability, translation efficiency, and cellular adaptation programs, RNA modifications may influence how AT2 cells respond to injury-associated stress [38]. However, current evidence remains insufficient to determine whether RNA modifications directly regulate AT2 lineage progression or primarily modulate the cellular conditions supporting epithelial regeneration.

### 4.2. Transitional Epithelial Populations During Aberrant Repair

Recent studies have identified KRT8^+^ transitional epithelial populations as intermediate epithelial populations associated with alveolar epithelial regeneration. These populations display characteristics of both activated AT2 cells and differentiating epithelial cells, reflecting the dynamic plasticity of alveolar epithelial cells during repair.

Rather than representing a terminally differentiated population, KRT8^+^ transitional cells are considered part of a continuum of epithelial populations that emerge during injury response and regeneration [3,4].

In fibrotic lungs, KRT8^+^ transitional epithelial populations appear to persist or accumulate under conditions of chronic epithelial stress. Single-cell transcriptomic studies have identified enrichment of these injury-associated transitional populations in fibrotic lungs, suggesting altered progression toward mature epithelial populations during disease development [39,40].

Spatial transcriptomic analyses have further revealed that KRT8^+^ transitional epithelial populations are preferentially localized within fibrotic regions and associated with altered interactions between epithelial and surrounding cellular compartments, suggesting that their persistence may reflect changes in the local repair environment [41].

These findings suggest that persistence of transitional epithelial populations may reflect incomplete progression along normal regenerative trajectories. Although emerging studies indicate that these populations may influence local epithelial–mesenchymal communication, current evidence remains insufficient to establish KRT8^+^ transitional populations as independent drivers of fibrosis. Instead, they are currently regarded as indicators of unresolved epithelial repair with potential functional contributions that require further validation.

Importantly, the functional interpretation of KRT8^+^ transitional epithelial populations remains under active investigation. Although their accumulation is consistently observed in fibrotic tissues and correlates with epithelial dysfunction, current evidence does not establish that these populations directly drive fibrosis progression. Instead, they are more appropriately regarded as populations associated with maladaptive epithelial repair and persistent injury-associated responses, with their potential functional contribution requiring further validation. Understanding the regulatory mechanisms controlling the formation and resolution of these transitional states may provide insights into how epithelial repair becomes dysregulated during fibrosis.

### 4.3. RNA Modifications as Potential Regulators of AT2 Repair Responses

Within the context of AT2 transition during repair, RNA modifications may function as modulators of epithelial adaptation by regulating transcript stability, translation efficiency, and stress-responsive gene expression. Unlike permanent genomic alterations, epitranscriptomic regulation allows dynamic adjustment of RNA output, enabling cells to adapt to changing environmental conditions during injury and repair [42].

Among currently studied RNA modifications, m6A modification has received the most attention in pulmonary epithelial biology. Experimental studies using primary alveolar epithelial cells have demonstrated that altered m6A regulation can influence epithelial responses to injury [38]. For example, METTL3-mediated m6A modification was shown to regulate PTEN mRNA stability and translation efficiency in AECII cells exposed to LPS stimulation, thereby affecting epithelial proliferation, viability, and inflammatory responses [38].

These findings provide experimental evidence that altered RNA modification can influence functional responses of alveolar epithelial cells during injury [38]. However, whether m6A or other RNA modifications directly regulate AT2-to-AT1 differentiation or influence the persistence of KRT8^+^ transitional epithelial populations remains unclear [43].

Current evidence mainly supports RNA modifications as modulators of epithelial stress adaptation, survival, and repair capacity rather than definitive determinants of epithelial cell states [42,43]. Future studies using AT2-specific genetic models, lineage-resolved approaches, and transcriptome-wide modification profiling will be required to clarify how RNA modifications influence epithelial transitions and repair outcomes in pulmonary fibrosis [44,45].

## 5. AT2 Cell Senescence and RNA Modification-Mediated Regulation of Aberrant Repair

Persistent alveolar epithelial injury can induce stable alterations in AT2 cell characteristics, including cellular senescence, impaired regenerative capacity, and abnormal secretory activity. Senescent AT2 cells exhibit characteristics distinct from transient stress responses, including persistent cell-cycle arrest, metabolic remodeling, and acquisition of senescence-associated secretory phenotypes (SASP), which may influence epithelial repair and the surrounding microenvironment during pulmonary fibrosis [42,43].

Unlike acute epithelial stress that can be resolved through adaptive repair responses, persistent senescence represents a maladaptive epithelial state associated with prolonged injury exposure. Increasing evidence suggests that AT2 senescence may contribute to defective regeneration by limiting epithelial proliferative potential and disrupting normal repair trajectories, thereby facilitating the persistence of abnormal epithelial states in fibrotic lungs [42,43].

### 5.1. Mechanisms of AT2 Senescence Induction

AT2 cell senescence is induced by multiple stress signals present in fibrotic lung environments, including endoplasmic reticulum (ER) stress, oxidative damage, mitochondrial dysfunction, and altered proteostasis. Genetic and experimental models have demonstrated that AT2-specific stress responses can initiate epithelial dysfunction and promote fibrotic remodeling. For example, SFTPC BRICHOS domain mutations induce AT2 ER stress and cytokine production, providing direct evidence that epithelial stress responses can contribute to lung fibrosis development [44].

In addition to ER stress, oxidative damage and regulated cell death pathways can further compromise AT2 cell function. Ferroptosis-associated mechanisms have been implicated in AT2 injury during pulmonary fibrosis, with dysregulation of oxidative metabolism and lipid homeostasis influencing epithelial vulnerability under fibrotic stress conditions [45].

Under persistent stress conditions, AT2 cells may activate classical senescence-associated regulatory pathways, including p53-p21 and p16-Rb signaling, resulting in stable proliferative arrest and reduced regenerative capacity. Such alterations may impair the ability of AT2 cells to participate in effective epithelial restoration after injury [46].

Importantly, AT2 senescence should not be interpreted as an independent initiating factor of fibrosis. Rather, it represents a stress-associated epithelial state that reflects the cumulative effects of chronic injury and may contribute to fibrosis by limiting epithelial repair capacity and modifying local cellular interactions. Emerging evidence further suggests that epitranscriptomic regulation may influence senescence-associated programs by modulating stress-responsive transcripts and RNA fate control [42].

### 5.2. SASP-Mediated Microenvironmental Remodeling

A major feature of cellular senescence is the acquisition of a senescence-associated secretory phenotype (SASP), characterized by increased production of inflammatory cytokines, chemokines, and tissue-remodeling factors. In the context of pulmonary fibrosis, SASP production by dysfunctional epithelial cells may provide a mechanism through which intracellular senescence states influence extracellular microenvironmental changes [47].

Senescent epithelial cells can release inflammatory mediators that affect neighboring immune and stromal populations, thereby promoting persistent inflammatory signaling and altered epithelial–mesenchymal communication. Changes in epithelial-derived signaling molecules may influence inflammatory communication and extracellular remodeling within the injured lung microenvironment [47,48].

In addition, impaired AT2 regeneration itself may contribute to abnormal tissue remodeling. Studies investigating alveolar progenitor regulation have shown that alterations in AT2 proliferation and self-renewal pathways influence the efficiency of epithelial recovery following injury, suggesting that failure of epithelial renewal is closely associated with defective repair processes [48].

However, the contribution of SASP to fibrosis may vary depending on the pathological context. SASP-associated factors may not directly initiate fibrosis but may amplify pre-existing injury responses by sustaining inflammatory communication and disrupting the balance between epithelial repair and pathological remodeling [49].

### 5.3. RNA Modification-Associated Regulation of Senescence Programs

RNA modifications represent an additional post-transcriptional regulatory layer that may influence senescence-associated programs by controlling RNA stability, translation efficiency, and stress-responsive gene expression. Among different RNA modifications, m6A has been most extensively investigated in fibrotic diseases because of its dynamic regulation and broad effects on cellular responses [42].

Recent studies have provided evidence linking m6A dysregulation with epithelial injury and senescence-associated processes. METTL3-mediated m6A modification has been shown to regulate alveolar epithelial cell responses after injury, affecting cellular viability, proliferation, and inflammatory responses, suggesting that RNA methylation may participate in controlling epithelial adaptation during stress conditions [43].

More specifically, METTL14-mediated m6A modification of DDIT4 transcripts has been implicated in aging-related idiopathic pulmonary fibrosis. Using IPF samples, MeRIP-seq, RNA sequencing, and experimental validation, this study demonstrated altered m6A patterns in IPF and linked METTL14-DDIT4 regulation with alveolar epithelial senescence-associated responses [44].

Beyond individual target transcripts, altered RNA modification patterns may influence broader stress-response networks associated with epithelial aging and fibrosis. However, current evidence remains largely focused on m6A, and whether other RNA modifications contribute to AT2 senescence-associated responses remains unclear [45,46].

Emerging single-cell RNA modification profiling technologies have further revealed substantial heterogeneity of m6A patterns among individual cells, suggesting that epitranscriptomic regulation may contribute to cellular state diversity within complex tissues. Such approaches may provide future opportunities to identify cell-type-specific RNA modification signatures associated with epithelial senescence during pulmonary fibrosis [47,50].

Overall, current evidence suggests that RNA modifications may influence AT2 senescence-associated responses through effects on stress adaptation and transcript regulation. However, whether specific RNA modification events directly regulate the transition from reversible epithelial stress responses to persistent senescence remains to be clarified [48,49].

## 6. RNA Modification-Mediated Regulation of Epithelial Plasticity and Repair Failure

Building upon the epithelial changes described above, this section focuses on the mechanisms underlying failed repair resolution rather than the emergence of transitional epithelial populations themselves. Although AT2-to-AT1 differentiation represents an essential component of alveolar regeneration, this regenerative trajectory may become disrupted under persistent fibrotic stress. Instead of efficiently progressing toward mature AT1 phenotypes, injured epithelial cells may remain in prolonged transitional populations characterized by incomplete maturation and altered repair programs. Impaired AT2-to-AT1 differentiation has been identified as an important feature of defective epithelial repair in fibrotic conditions [19].

Beyond transcriptional dysregulation, post-transcriptional regulatory mechanisms may contribute to the failure of repair resolution. RNA modifications provide an additional regulatory layer that can influence transcript stability, processing, localization, and translation efficiency, thereby shaping cellular responses under injury conditions [37].

### 6.1. RNA Modification-Mediated Regulation of Epithelial Plasticity

AT2-to-AT1 differentiation requires coordinated regulation of developmental signals and epithelial identity programs. Previous studies have identified multiple regulatory pathways controlling epithelial maturation and regeneration [3,4]. For example, thyroid hormone receptor β activation promotes AT2-to-AT1 differentiation through regulation of KLF2 and CEBPA, enhancing alveolar regeneration after injury [30].

The maintenance of epithelial progenitor characteristics and appropriate transition toward differentiated states also depends on precise regulation of cellular identity programs. Disruption of these regulatory networks may disturb the balance between epithelial maintenance and differentiation, contributing to defective repair responses [31].

RNA modifications may provide an additional regulatory mechanism controlling epithelial plasticity by influencing transcript stability and translation efficiency. Through modulation of RNA output, these modifications may affect cellular adaptation programs during injury and repair [37,38].

Among various RNA modifications, m6A is currently the most extensively investigated modification in pulmonary fibrosis-related research. Experimental evidence indicates that m6A-related regulators participate in fibrosis-associated cellular responses by affecting RNA metabolism and downstream biological functions, suggesting a potential role in pathological cell-state transitions [38,40].

However, current evidence remains insufficient to conclude that RNA modifications directly determine AT2 lineage fate. Instead, they are more appropriately considered modulators that influence epithelial adaptation, stress responses, and repair capacity [37,40].

### 6.2. Persistence of Injury-Associated Transitional Epithelial Programs in Fibrotic Lungs

Failure of epithelial repair resolution represents a major feature of pulmonary fibrosis. Under persistent injury conditions, transitional epithelial programs may fail to terminate appropriately and become prolonged components of abnormal repair landscapes [29,40].

Rather than representing independent pathogenic cell populations, persistent transitional epithelial populations are increasingly interpreted as indicators of unresolved epithelial stress and incomplete regeneration. Their accumulation may reflect the inability of injured epithelial cells to successfully complete normal repair trajectories [29,30].

The persistence of injury-associated epithelial programs is likely regulated by interactions between intrinsic epithelial responses and surrounding microenvironmental signals. Recent studies have demonstrated that fibrotic epithelial states are closely associated with specific immune and stromal niches, suggesting that epithelial dysfunction develops within a complex multicellular environment [51,52].

In addition, profibrotic signals within the tissue microenvironment may reinforce epithelial stress responses and contribute to maintenance of transitional states. Such feedback interactions may provide a potential explanation for why injury-associated epithelial programs remain unresolved during chronic fibrosis [52,53].

Nevertheless, whether persistent transitional states actively promote fibrosis progression or primarily represent consequences of unresolved epithelial injury remains unclear. Further lineage-tracing and cell-type-specific functional studies are required to define their precise biological roles [3,4].

### 6.3. Potential RNA Regulatory Mechanisms Underlying Repair Failure

Beyond alterations in cellular states, increasing evidence suggests that RNA regulatory mechanisms may contribute to the failure of repair resolution by modulating stress adaptation and transcript output [37,38].

Among various RNA modifications, m6A is currently the most extensively characterized modification in pulmonary fibrosis-related research. Experimental studies have demonstrated that m6A-related regulators participate in fibrosis-associated cellular responses by altering transcript fate and downstream signaling pathways, suggesting a potential role in regulating pathological cell-state transitions [38,40].

Beyond m6A, emerging evidence suggests that other RNA modifications may contribute to cellular adaptation under pathological conditions. m5C modification has been implicated in regulating RNA stability and functional responses in disease contexts, although its specific role in alveolar epithelial repair remains largely unknown [54].

Similarly, m1A and m7G modifications have been reported to influence RNA metabolism, translation efficiency, and cellular homeostasis in various biological systems [55,56]. These findings suggest that non-m6A modifications may represent additional regulatory layers involved in epithelial stress responses, but direct evidence in AT2 cells remains limited [55,56].

RNA editing represents another important post-transcriptional regulatory mechanism. A-to-I RNA editing mediated by ADAR enzymes can alter RNA sequences, RNA structures, and immune-related signaling pathways, providing a potential link between cellular stress responses and altered gene regulation [57].

Importantly, current evidence linking specific RNA modifications directly to persistent transitional epithelial states or maladaptive repair programs remains insufficient. Therefore, RNA modifications should currently be considered potential regulators influencing epithelial plasticity, stress adaptation, and repair resolution rather than established determinants of epithelial fate [58,59].

### 6.4. Current Evidence Gaps in RNA Modification-Mediated Epithelial Repair Regulation

Despite increasing interest in RNA modifications in pulmonary fibrosis, several important questions remain unresolved regarding their roles in alveolar epithelial repair [1,10]. First, many studies have relied on bulk tissue analysis or non-cell-type-specific models, making it difficult to determine whether observed RNA modification changes originate from AT2 cells or other cellular populations [56].

Second, although m6A-related mechanisms have been increasingly investigated, direct evidence connecting RNA modifications with AT2-specific differentiation failure, transitional state persistence, or epithelial repair resolution remains limited. Future studies using cell-type-specific models are required to clarify whether RNA modifications influence epithelial transitions or primarily represent secondary responses to injury [57,58].

Third, the functional consequences of RNA modifications at specific transcript sites remain insufficiently characterized. Spatial and transcriptomic studies have highlighted the importance of cellular niches and intercellular interactions in shaping fibrotic epithelial states, emphasizing the need to integrate molecular regulation with tissue context [53,55].

Furthermore, development of high-resolution RNA modification mapping technologies may provide opportunities to identify modification landscapes at transcript and cell-type levels. Integrating these approaches with single-cell sequencing and lineage-tracing strategies may help clarify whether RNA modifications contribute to epithelial repair outcomes or primarily reflect downstream consequences of cellular stress [55,59].

Overall, current evidence supports RNA modifications as potential regulators of epithelial plasticity and stress adaptation. However, their precise contribution to pulmonary fibrosis progression and epithelial repair failure requires further mechanistic validation [58,59].

## 7. RNA Modifications Regulate Epithelial–Mesenchymal Communication and Fibrotic Niche Formation

Beyond regulating epithelial intrinsic responses, RNA modifications may also influence how injured epithelial states interact with surrounding stromal and immune populations, thereby extending their regulatory effects from intracellular adaptation to tissue-level communication. In this context, epithelial–mesenchymal communication represents an additional layer through which altered epithelial states contribute to the establishment and persistence of fibrotic niches.

### 7.1. Aberrant Epithelial States Reshape the Fibrotic Microenvironment

Pulmonary fibrosis is increasingly recognized as a disorder of disrupted multicellular communication, in which persistent abnormalities of alveolar epithelial responses reshape interactions with mesenchymal and immune compartments. Rather than being solely a consequence of fibroblast activation, fibrotic remodeling involves reciprocal communication networks in which injured epithelial cells and surrounding stromal populations continuously influence each other.

During normal alveolar repair, AT2 cells function as facultative progenitors that undergo activation, transitional remodeling, and differentiation toward AT1 cells to restore epithelial integrity. Multiple studies have demonstrated that this regenerative trajectory is tightly regulated by lineage-associated factors, including Fgfr2- and Tfcp2l1-dependent programs, which contribute to AT2 cell fate maintenance and differentiation during alveolar regeneration [60,61]. However, persistent injury conditions may disrupt these epithelial state transitions, resulting in accumulation of intermediate populations with incomplete maturation characteristics [39].

Single-cell transcriptomic analyses have identified distinct transitional epithelial populations in fibrotic lungs, including KRT8^+^ injury-associated states that exhibit features of stress adaptation and altered differentiation programs. Rather than representing isolated cellular abnormalities, these transitional epithelial populations may participate in remodeling of local intercellular communication networks by altering interactions between epithelial, stromal, and immune compartments within fibrotic niches [39,62].

Importantly, these populations should not be interpreted as independent pathogenic lineages; rather, they may represent context-dependent epithelial states reflecting unresolved repair responses and altered communication between epithelial and mesenchymal compartments [63].

Spatial transcriptomic studies further indicate that fibrotic lesions contain highly organized cellular niches in which epithelial, stromal, and immune populations are spatially coordinated. Such pathological niches provide a microenvironment in which abnormal epithelial states may interact with fibroblasts and inflammatory cells, thereby reinforcing maladaptive repair processes [64,65].

Recent functional studies have provided direct evidence that abnormal epithelial intermediates can influence fibroblast behavior. Aberrant intermediate alveolar epithelial cells have been shown to establish pathogenic interactions with lung fibroblasts and promote fibroblast activation, suggesting that persistent epithelial abnormalities may contribute to remodeling of the surrounding stromal environment through altered signaling outputs [39].

Together, these findings support a model in which persistent epithelial abnormalities represent important components of the fibrotic communication network. Although these states are not necessarily primary drivers of fibrosis, their persistence may alter epithelial–mesenchymal interactions and contribute to a microenvironment that favors chronic remodeling.

### 7.2. Reciprocal Epithelial–Fibroblast Signaling and Inflammatory Niche Formation

Persistent epithelial injury is associated not only with failed regeneration but also with altered signaling environments that may influence neighboring stromal and immune populations. Through the release of soluble mediators, extracellular vesicles, and inflammatory factors, dysfunctional epithelial cells may alter surrounding cellular behaviors and contribute to maintenance of a fibrotic niche [66].

Senescence-associated alterations in alveolar epithelial cells represent an important mechanism linking epithelial dysfunction with inflammatory communication. During chronic injury, AT2 cells may acquire senescence-associated secretory phenotypes (SASP), characterized by increased production of inflammatory cytokines, chemokines, and tissue-remodeling factors [67]. These secretory programs provide a potential mechanism through which senescent epithelial states influence macrophage polarization and fibroblast activation during fibrotic remodeling [68,69].

Experimental studies have demonstrated that senescent epithelial cells can regulate immune and stromal responses through specific secretory mediators. Senescence-associated pathways involving PAI-1 signaling have been implicated in regulating epithelial inflammatory phenotypes and extracellular remodeling responses, suggesting that senescent epithelial states may actively influence the surrounding microenvironment [70,71].

In addition, PAI-1-related signaling has been shown to modulate p53 activation, SASP production, and macrophage responses, providing a potential mechanism linking epithelial senescence with inflammatory niche formation [72].

The interaction between epithelial cells and fibroblasts is likely bidirectional. Fibroblast-derived signals can further reshape epithelial repair trajectories by altering progenitor activation and differentiation programs [73]. Recent studies using human lung tissue, epithelial–fibroblast co-culture organoids, and precision-cut lung slices demonstrated that fibroblast-associated TGF-β1 signaling promotes epithelial metaplastic remodeling through an sFRP2-mediated noncanonical Wnt pathway, highlighting a mechanism by which stromal signals influence epithelial states during fibrotic progression [74].

Cytokine-mediated communication represents another important component of epithelial–mesenchymal feedback. IL-11, a cytokine implicated in fibrotic remodeling, has been shown to impair alveolar epithelial progenitor function [75]. Using mouse lung organoids and precision-cut lung slice models, IL-11 exposure reduced epithelial progenitor activation and suppressed the formation of mature alveolar epithelial populations, suggesting that inflammatory signaling within the fibrotic microenvironment can directly interfere with epithelial regeneration [75].

Extracellular vesicle-mediated communication provides an additional mechanism for transferring regulatory information between epithelial and mesenchymal compartments. Exosomes derived from injured or stressed cells contain diverse molecular cargos, including proteins, lipids, and RNA species, which may alter recipient cell behavior and influence inflammatory or fibrotic responses [76]. However, whether RNA modification-dependent regulation of extracellular vesicle cargo directly contributes to pulmonary fibrosis remains insufficiently understood [77]. This represents a potential link between epitranscriptomic regulation and intercellular communication, although direct evidence in alveolar epithelial systems remains limited [77].

Overall, epithelial–mesenchymal communication in pulmonary fibrosis should be considered a dynamic feedback process rather than a linear signaling pathway. Persistent epithelial stress, senescence-associated secretion, cytokine signaling, and stromal activation collectively contribute to maintenance of a pathological niche in which repair processes remain unresolved [78].

### 7.3. RNA Modifications as Regulatory Layers of Epithelial–Mesenchymal Communication

RNA modifications provide an additional layer of post-transcriptional regulation that may influence epithelial–mesenchymal communication by shaping RNA stability, translation efficiency, and stress-responsive gene expression [10]. Unlike classical signaling pathways that transmit extracellular information through defined molecular cascades, epitranscriptomic regulation may fine-tune the magnitude, duration, and context dependence of cellular responses during chronic injury [79].

Among currently characterized RNA modifications, m6A has received the most attention because of its dynamic regulation and broad effects on RNA metabolism [80]. Increasing evidence indicates that m6A-related regulators participate in cellular stress adaptation, inflammatory responses, and senescence-associated transcriptional programs, suggesting that altered RNA methylation may influence the communication outputs of injured epithelial and stromal cells [79,80].

A growing body of evidence suggests that m6A regulation is closely associated with senescence-related secretory programs [80]. Studies have demonstrated that m6A regulators influence inflammatory transcript expression and SASP-related programs during cellular senescence, suggesting that RNA modification may contribute to altered secretory phenotypes of senescent cells [80,81].

In fibroblast senescence models, m6A remodeling has been shown to regulate the stability of SASP-associated transcripts, providing additional evidence that epitranscriptomic regulation may influence communication between senescent stromal cells and neighboring populations [81].

In alveolar epithelial biology, altered m6A regulation has also been linked to cellular stress responses [79]. Experimental studies have demonstrated that m6A-related pathways can regulate oxidative stress adaptation and injury responses in pulmonary epithelial cells, suggesting that RNA modification may influence epithelial states that subsequently affect intercellular signaling networks [79,82].

Analysis of human pulmonary fibrosis datasets further suggests that m6A regulatory factors are altered in fibrotic lung tissues, indicating potential associations between epitranscriptomic dysregulation and disease-associated cellular states [80]. However, whether altered m6A patterns directly reshape epithelial–mesenchymal communication requires further cell-type-specific validation [80].

Beyond m6A, other RNA modifications may also contribute to regulation of cellular adaptation and communication [81]. m5C modification has been implicated in RNA stability, translation regulation, and stress responses, although its specific role in pulmonary epithelial–mesenchymal interactions remains largely unexplored [81]. Emerging studies have also suggested that RNA modification dynamics may influence progenitor differentiation programs, providing a potential link between epitranscriptomic regulation and epithelial plasticity during tissue repair [82].

Studies from other epithelial systems further indicate that RNA modification-dependent regulation of developmental pathways, including Notch-associated programs, may influence cellular plasticity and differentiation states [82]. However, whether these mechanisms operate in pulmonary fibrosis remains uncertain and requires direct investigation [82].

Among non-m6A RNA regulatory mechanisms, RNA editing represents another potential layer influencing inflammatory communication [82]. ADAR1-mediated A-to-I editing regulates double-stranded RNA recognition and innate immune signaling, suggesting that RNA editing may modulate epithelial stress responses and inflammatory states [82]. Nevertheless, direct evidence connecting RNA editing alterations with epithelial–mesenchymal communication in pulmonary fibrosis remains limited [82].

Evidence linking RNA modification to fibrosis-associated signaling has also been provided by studies of ac4C modification [83]. NAT10-mediated ac4C modification has been shown to regulate fibrosis-associated RNA programs and may influence pulmonary fibrotic responses, providing a specific example of how epitranscriptomic regulation can affect pathological signaling outputs [83]. However, whether such mechanisms directly reshape epithelial–mesenchymal communication remains to be determined [83].

Collectively, current evidence suggests that RNA modifications may function as potential regulators of epithelial–mesenchymal communication by modulating the stability and functional output of injury-associated transcriptional programs, while their contribution to fibrosis may vary across different biological contexts.

## 8. Non-m6A RNA Modifications in Pulmonary Fibrosis

Compared with m6A, the functional understanding of non-m6A RNA modifications in pulmonary fibrosis remains relatively limited. Current evidence is mainly derived from profiling studies, whole-lung tissue analyses, non-alveolar models, or indirect functional investigations, and AT2 cell-specific validation remains insufficient [82,84]. Although non-m6A modifications have been implicated in RNA stability, translational regulation, immune sensing, and stress adaptation, their specific roles in alveolar epithelial injury and aberrant repair remain incompletely understood [20,84].

Among non-m6A modifications, m5C has been relatively better characterized in fibrosis-related contexts, whereas evidence regarding m1A, m7G, pseudouridine (Ψ), and A-to-I RNA editing in pulmonary fibrosis remains limited, particularly regarding cell-type-specific mechanisms and functional validation [18,82]. Therefore, non-m6A modifications are currently better viewed as emerging regulatory layers that may influence epithelial stress responses and repair-associated processes rather than established drivers of pulmonary fibrosis [82,84].

### 8.1. m5C RNA Modification

5-methylcytidine (m5C) is an RNA modification involved in post-transcriptional regulation, particularly through effects on RNA stability and RNA-protein interactions [20,32]. Compared with other non-m6A modifications, m5C has been relatively better investigated in fibrosis-associated conditions [18,82]. Recent studies have reported altered m5C modification patterns in pulmonary fibrosis models, suggesting that m5C may participate in fibrosis-associated stress responses through regulation of RNA stability and related post-transcriptional processes [18].

Mechanistically, m5C deposition is mediated mainly by NSUN family methyltransferases, while modified transcripts can interact with RNA-binding proteins such as YBX1 and ALYREF, thereby influencing RNA fate and post-transcriptional regulation [20,32]. However, current evidence linking m5C to pulmonary fibrosis remains largely based on profiling analyses or non-cell-specific models [82]. Whether m5C directly regulates AT2 dysfunction, epithelial transitions, or abnormal repair processes requires further validation [82].

Therefore, m5C is more appropriately considered an emerging post-transcriptional regulatory layer that may influence epithelial stress responses rather than an independent pathogenic pathway [84].

### 8.2. m1A RNA Modification

N1-methyladenosine (m1A) has been implicated in RNA structure regulation, translation control, and stress-associated RNA metabolism [84]. However, direct evidence linking m1A to pulmonary fibrosis or AT2-specific responses remains limited [82].

Given the importance of metabolic adaptation and mitochondrial regulation in alveolar epithelial injury, m1A may represent a potential stress-associated regulatory layer involved in cellular adaptation [84]. However, whether m1A contributes to AT2 dysfunction or abnormal epithelial repair remains largely unknown and requires validation in disease-relevant models [82].

### 8.3. m7G and Pseudouridine Modifications: Emerging Evidence

m7G modification has been implicated in RNA stability and translational regulation through METTL1/WDR4-dependent pathways [84]. Although direct evidence linking m7G to pulmonary fibrosis remains limited, m7G-associated RNA regulation may represent a potential mechanism involved in epithelial stress adaptation [84]. Pseudouridine (Ψ) modification contributes to RNA structural regulation and translational control; however, its specific role in pulmonary fibrosis, particularly in alveolar epithelial systems, remains largely unexplored [84].

Considering the limited evidence currently available, m7G and Ψ should be regarded as potential regulatory layers requiring further investigation, particularly in AT2-specific fibrotic models [82].

### 8.4. A-to-I RNA Editing

A-to-I RNA editing represents an ADAR-dependent RNA regulatory process involved in RNA structure modulation and immune sensing [83,85]. By regulating endogenous double-stranded RNA recognition, ADAR-mediated editing contributes to innate immune regulation and cellular stress responses [83].

Among ADAR family members, ADAR1 has been reported to regulate dsRNA sensing through pathways involving MDA5, PKR, and ZBP1, thereby influencing interferon-associated immune responses [83]. These functions suggest that A-to-I editing may participate in inflammatory stress regulation within injured tissues [86].

In fibrotic conditions, dysregulated ADAR-mediated editing has been associated with inflammatory responses and tissue remodeling [86,87]. However, direct evidence demonstrating AT2-specific roles of A-to-I editing in pulmonary fibrosis remains limited [82]. Future studies are needed to clarify whether ADAR-dependent RNA editing contributes to AT2 dysfunction, transitional epithelial states, or abnormal repair responses [85,87].

### 8.5. Integrated Perspective

Collectively, non-m6A RNA modifications may represent complementary regulatory layers that influence pulmonary epithelial responses during injury and repair [82,84]. Compared with m6A, which currently has relatively stronger mechanistic evidence in pulmonary fibrosis, most non-m6A modifications remain at an early stage of investigation [82].

Current findings suggest that non-m6A modifications may affect RNA stability, translational regulation, immune sensing, and stress adaptation; however, their epithelial-specific functions and functional relevance to fibrotic progression remain unclear [20,84].

**Figure 2:**
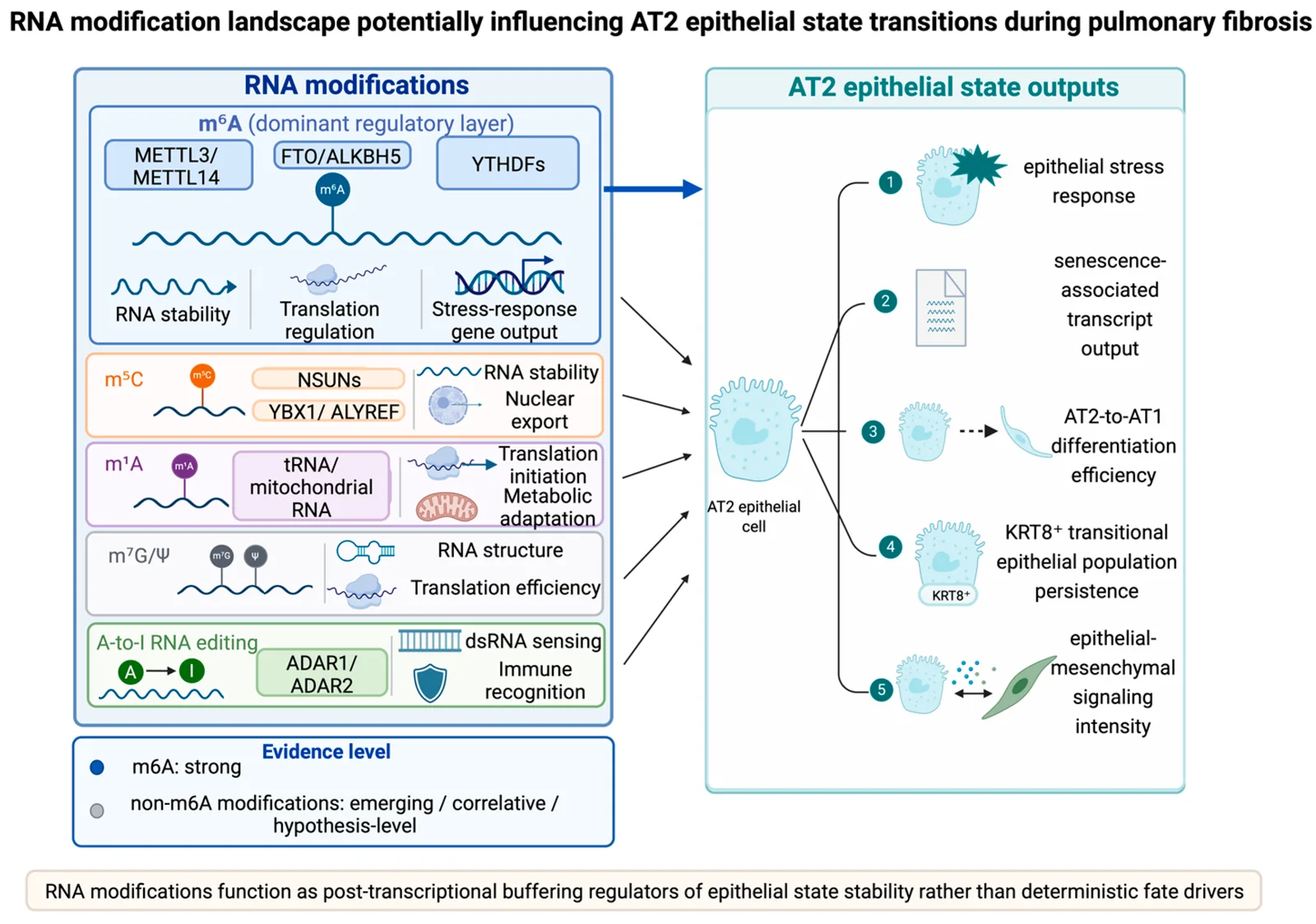
RNA modification landscape potentially influencing AT2 epithelial state transitions during pulmonary fibrosis. Major RNA modification layers, including m6A, m5C, m1A, m7G/Ψ, and A-to-I RNA editing, may regulate RNA stability, translation efficiency, stress-response transcript output, and immune recognition, thereby influencing AT2 epithelial stress responses, senescence-associated transcript remodeling, AT2-to-AT1 differentiation efficiency, KRT8^+^ state persistence, and epithelial–mesenchymal signaling plasticity. Solid arrows indicate potential regulatory relationships, whereas dashed arrows represent potential indirect associations or transitional processes. Different colors distinguish individual RNA modification categories and evidence levels, with m6A representing the most extensively characterized modification and non-m6A modifications representing emerging regulatory layers with limited functional validation.

Future studies integrating multi-omics approaches, AT2-specific genetic models, lineage-tracing strategies, and site-level functional validation will be important to clarify the roles of non-m6A modifications in pulmonary fibrosis, especially for determining whether specific RNA modifications directly influence AT2 repair responses [82,84] (Table 2) (Figure 2).

## 9. Technical Systems and Experimental Strategy

Current RNA modification studies in pulmonary fibrosis face several unresolved challenges, including limited cell-type specificity, insufficient site-level characterization, and inadequate functional validation. Because alveolar epithelial injury and repair failure involve dynamic interactions among multiple epithelial populations, future studies should address three fundamental questions: which cell populations exhibit disease-associated RNA modification changes, which specific RNA modification events are functionally relevant, and whether these modifications produce measurable biological consequences during epithelial repair.

**Figure 3:**
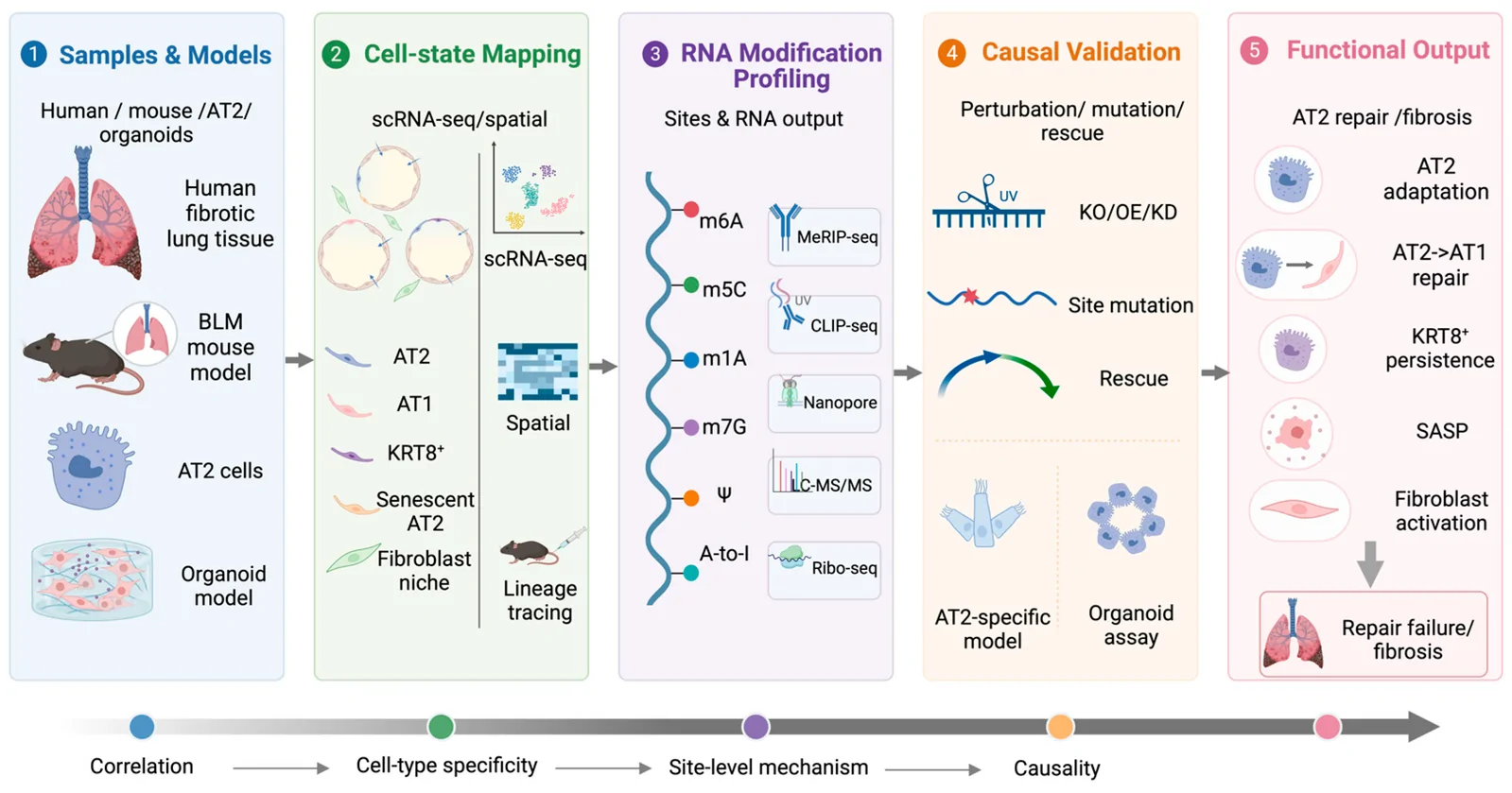
Multi-level experimental strategy for functional validation of RNA modification-associated alveolar epithelial repair failure in pulmonary fibrosis. Human fibrotic lung tissues, experimental fibrosis models, primary AT2 cells, and organoid-based systems provide complementary platforms for studying epithelial repair failure. Cell-type-resolved approaches, including single-cell and spatial analyses, enable characterization of AT2 cells, AT1 cells, KRT8^+^ transitional epithelial populations, senescent epithelial populations, and fibroblast-associated niches. RNA modification profiling identifies candidate modification events and associated RNA changes, whereas functional validation requires AT2-specific perturbation, site-level modification analysis, and rescue approaches. The colored boxes represent different experimental stages, including sample and model selection, cell-state mapping, RNA modification profiling, causal validation, and functional outcome assessment. Arrows indicate the sequential progression between experimental stages. The gradient scale at the bottom represents increasing evidence resolution from correlation to cell-type specificity, site-level mechanism, and causality.

Addressing these questions requires integration of human fibrotic lung samples, experimental fibrosis models, and AT2-relevant in vitro systems [40]. Cell-type-resolved approaches can identify RNA modification patterns associated with specific epithelial populations, whereas site-level mapping and functional perturbation strategies are required to determine whether candidate modifications influence RNA stability, translation, or downstream repair phenotypes [88,89]. Therefore, integration of single-cell approaches, spatial profiling, modification-specific detection, and genetic validation may help move the field beyond descriptive associations toward functionally supported evidence [90,91] (Figure 3). 

### 9.1. AT2-Relevant Models for Cell-Specific Validation

A major limitation in current RNA modification research in pulmonary fibrosis is the lack of AT2 cell-specific functional validation. Although in vivo fibrosis models, such as bleomycin-induced pulmonary fibrosis, reproduce important pathological features including epithelial injury, senescence, and impaired repair, these models involve complex interactions among epithelial, immune, and stromal compartments, making it difficult to distinguish AT2 cell-intrinsic effects from secondary responses [40,57].

Therefore, future studies should prioritize AT2-relevant experimental systems, including AT2-specific genetic models, primary AT2 cell cultures, alveolar organoid systems, and induced pluripotent stem cell (iPSC)-derived alveolar epithelial models [40,92]. These platforms provide opportunities to determine whether RNA modification changes influence epithelial repair capacity rather than simply reflect tissue-level alterations [88,91]. Human pluripotent stem cell-derived alveolar organoids have been developed as disease-relevant systems for modeling epithelial dysfunction and evaluating potential therapeutic strategies [40,92].

In addition, emerging delivery approaches, such as inhalable mRNA-lipid nanoparticle systems, provide potential tools for testing whether modulation of epithelial regulatory programs can restore AT2 regenerative capacity. However, their efficiency, specificity, and safety require further validation before clinical translation [93,94].

### 9.2. Cell-Type and Site-Level Mapping of RNA Modification Dynamics

Aberrant alveolar repair involves dynamic changes in epithelial populations rather than simple alterations in cell abundance. In particular, the emergence and persistence of KRT8^+^ transitional epithelial populations represent important features of failed epithelial regeneration, although their relationship with RNA modification regulation remains incompletely understood [37,66].

Bulk transcriptomic and RNA modification analyses provide valuable information regarding global changes; however, they cannot determine whether specific modification events occur within defined epithelial populations. Therefore, integration of single-cell transcriptomics, spatial transcriptomics, and cell-type-resolved RNA modification profiling is required to identify modification patterns associated with specific epithelial populations and their surrounding microenvironments.

Single-cell approaches can resolve cellular heterogeneity during injury and repair, whereas spatial technologies provide information regarding the localization of transitional epithelial populations and their interactions with neighboring stromal or immune cells. Recent studies have demonstrated the importance of resolving intermediate epithelial populations during lung regeneration and fibrosis progression [66].

For RNA modification research, these approaches are particularly important because many current m6A-related studies rely on bulk tissue measurements, which may obscure cell-specific regulatory effects [88,95]. Emerging single-cell RNA modification profiling approaches provide opportunities to determine whether candidate modifications occur preferentially in AT2 cells, transitional epithelial populations, or other cellular compartments. Recent advances in single-cell m6A profiling further demonstrate the feasibility of resolving RNA modification patterns at cellular resolution, although their application in pulmonary fibrosis remains limited [90,92].

### 9.3. Functional Validation of Candidate RNA Modification Events

A major challenge in current RNA modification research is distinguishing functional regulatory events from secondary alterations associated with tissue injury. Therefore, future studies should move beyond differential modification profiling and determine whether specific RNA modification events produce measurable biological effects.

Functional validation should include genetic perturbation of RNA modification regulators, site-specific modification analysis, mutation-based validation of candidate modification sites, and rescue experiments. These approaches are necessary to determine whether RNA modification changes influence target RNA stability, translation efficiency, protein output, and epithelial repair responses.

For m6A-centered studies, approaches such as DART-seq and other antibody-independent methods may improve site-level resolution and reduce limitations associated with antibody-based enrichment strategies [88,95]. Studies investigating RNA modification regulators in cellular stress responses have demonstrated that RNA modifications may participate in DNA damage responses and repair-associated processes; however, their specific contribution to AT2-mediated repair requires direct validation in relevant models [54,57].

Because different RNA modification detection methods provide distinct levels of information, experimental design should match the biological question. Global abundance measurements, transcriptome-wide modification mapping, candidate-site identification, and functional validation represent different evidence levels and should not be considered interchangeable [88,91].

### 9.4. KRT8^+^ Transitional Epithelial Populations and Experimental Interpretation

KRT8+ transitional epithelial populations have emerged as important indicators of abnormal alveolar repair. These populations appear during epithelial regeneration and accumulate under fibrotic conditions; however, their precise functional contribution remains incompletely understood and requires further investigation [57,66].

Current evidence supports the interpretation that KRT8^+^ transitional populations reflect incomplete epithelial differentiation and unresolved repair responses rather than universally acting as independent drivers of fibrosis. Therefore, experimental studies should distinguish between their identification, their association with impaired repair, and direct demonstration of functional effects.

RNA modifications may influence the formation, persistence, or resolution of these transitional epithelial populations by regulating stress-responsive transcripts, differentiation-associated RNA programs, or cellular adaptation processes. However, whether specific RNA modification events actively regulate KRT8^+^ population dynamics remains unclear because current evidence lacks sufficient AT2-specific and site-level functional validation [37,89].

Future investigations combining lineage-tracing models, AT2-specific manipulation, modification-site analysis, and functional rescue approaches will be necessary to clarify whether RNA modification pathways influence transitional epithelial population behavior during fibrosis progression and repair.

### 9.5. Integrated Experimental Perspective

Future RNA modification studies in pulmonary fibrosis should be organized around three interconnected questions: which cell populations exhibit disease-relevant RNA modification changes, which modification events regulate functional RNA outputs, and whether these molecular changes influence epithelial repair outcomes.

Answering these questions requires integration of AT2-relevant experimental systems, cell-type-resolved profiling, site-level modification analysis, and functional validation strategies. Such approaches may help clarify whether RNA modifications represent regulatory components associated with failed epithelial repair and determine their potential relevance for epithelial repair-centered therapeutic development [66,96].

In addition, standardized quantitative approaches, including LC–MS/MS-based measurement of global RNA modification abundance, may improve comparison across studies and provide complementary biochemical evidence for transcriptomic observations [88,94]. A summary of representative experimental models and multi-omics approaches for investigating RNA modification-associated AT2 injury and aberrant repair in pulmonary fibrosis is provided in Table 3. 

## 10. Translational Medical Value and Challenges

### 10.1. Biomarkers and Molecular Subtyping

Current clinical assessment of pulmonary fibrosis still primarily relies on high-resolution computed tomography (HRCT) and pulmonary function parameters, whereas RNA modification-related biomarkers may provide complementary molecular stratification if validated in independent cohorts [97]. Circulating extracellular vesicle-based biomarkers, including plasma exosomal circRNAs and serum EV proteins, may further support non-invasive IPF diagnosis and disease-activity assessment [98,99].

However, these approaches have limited capability in molecular-level identification of early alveolar epithelial injury and aberrant repair states [100,101]. With advances in epitranscriptomic research, RNA modification-related molecules are increasingly considered potential biomarkers, but their clinical translation requires robust feature selection, external validation, and mechanistic interpretation [97,102].

m6A-related regulatory factors are involved in epithelial stress, inflammatory output, and pro-fibrotic signaling regulation, while selected m6A regulators such as ALKBH5 have also been implicated in fibroblast activation in silica-induced pulmonary fibrosis [103]. Among them, the YTHDF1/m6A/NREP axis has been reported in alveolar epithelial cells in environmental exposure-related pulmonary fibrosis models [104]. Therefore, m6A-related molecules are more appropriately considered candidate biomarkers reflecting epithelial stress, aberrant repair states, and m6A regulator-defined risk patterns, rather than clinically established diagnostic markers [102,103].

Non-m6A modifications may also provide complementary information for disease subtyping, as m5C-associated gene signatures have been linked to diagnostic modeling and immune-related classification in IPF [105]. m5C and m1A can influence RNA stability, nuclear export, tRNA maturation, and translational processes, thereby providing mechanistic clues for their involvement in disease classification; in IPF cohorts, m5C-associated genes have also been used for diagnostic modeling and immune stratification [105,106]. m7G and Ψ are mainly associated with translational efficiency, RNA structural stability, and RNA–protein interactions, and their value as subtyping biomarkers still requires validation in pulmonary fibrosis samples [107,108].

In addition, KRT8^+^ transitional-state-related markers (such as CLDN4 and KRT17) can be used to reflect aberrant epithelial repair states [109,110]. Circulating EV-derived signatures, such as serum EV SFTPB, may further provide complementary information for epithelial state-oriented disease-activity assessment [98,111]. However, these markers still lack standardized clinical validation systems, including large-scale external cohorts, prospective performance evaluation, and clinically interpretable modeling pipelines [97,102].

### 10.2. Therapeutic Strategies and Potential Targets

Existing anti-fibrotic drugs (such as pirfenidone and nintedanib) can slow the decline in lung function but cannot reverse aberrant epithelial repair trajectories [112,113]. In recent years, novel agents such as nerandomilast have shown efficacy in reducing FVC decline in progressive pulmonary fibrosis; however, their therapeutic goal remains primarily disease progression delay [113,114].

Therefore, RNA modification regulatory networks may provide potential therapeutic opportunities by modulating epithelial stress responses and repair-associated RNA programs [115]. The m6A regulatory system has attracted therapeutic interest because selective inhibitors targeting METTL3 or FTO have provided proof-of-concept for pharmacological modulation of RNA-modifying enzymes [115,116]. This rationale is supported by the dynamic regulation of m6A through writers, erasers, and readers and by its broad effects on RNA processing, export, translation, and metabolism [115,116]. In pulmonary fibrosis, however, the translational relevance of m6A regulators remains dependent on identifying disease-relevant target RNAs, defining cell-type-specific effects, and validating therapeutic benefit and safety in pulmonary models; METTL14-mediated regulation of DDIT4 mRNA stability in aging-related IPF provides one potential disease-relevant example [104,116].

In pulmonary fibrosis models, the YTHDF1/m6A/NREP axis has been associated with epithelial-derived TGF-β1 signaling and fibroblast-to-myofibroblast transition, suggesting a potential target for modulating epithelial–mesenchymal communication [104,115]. In addition, METTL3-dependent m6A regulation of CXCL8 and ICAM-1 mRNA stability supports a broader role for m6A in inflammatory cell communication, suggesting that RNA modification factors may influence inflammation- and senescence-associated microenvironment remodeling [103,116].

Non-m6A modifications may eventually broaden intervention strategies; however, current evidence remains predominantly mechanistic or exploratory [105,107]. m5C and m1A have been linked to RNA stability and translational regulation, whereas direct therapeutic evidence for m7G and Ψ in pulmonary fibrosis remains unavailable [105,107].

Therefore, RNA modification-based interventions are currently more appropriately considered experimental strategies for modulating epithelial stress and repair responses rather than established clinical therapies, especially because existing mechanistic evidence for METTL3/TGFB1 or METTL3/β-catenin regulation remains largely derived from non-pulmonary or cross-organ fibrotic models [115,116]; Nevertheless, inhaled LNP-mediated mRNA antibody therapy targeting IL-11 provides preclinical evidence supporting the feasibility of local pulmonary mRNA delivery in experimental IPF-like fibrosis [113]. More broadly, inhalable mRNA-LNP delivery has also been used to promote epithelial regeneration by restoring AT2 stemness in experimental pulmonary fibrosis [114]. Their future application depends on AT2 cell-specific validation, target RNA identification, site-level functional characterization, and the development of safe delivery systems, particularly inhalable nucleic acid delivery platforms capable of overcoming nebulization stress, mucus barriers, and limited pulmonary transfection efficiency [93,117].

### 10.3. Translational Bottlenecks and Key Challenges

Although RNA modifications show potential translational relevance in pulmonary fibrosis, their clinical application remains constrained by substantial biological and technical limitations [117]. First, most current studies rely on whole-lung tissues, mixed-cell systems, or non-specific models, making it difficult to define AT2 cell origin and cell-autonomous effects [100,102].

Second, existing studies are largely limited to expression-based correlations, and a complete causal chain linking “modification site → target RNA → functional output” has not yet been established [89,117]. The functional role of KRT8^+^ transitional cells remains controversial, and whether they represent independent pathogenic drivers of fibrosis progression is still not fully resolved [109,110].

In addition, the functional evidence for non-m6A modifications in pulmonary fibrosis remains uneven [105]. Among them, m5C has shown some fibrosis-related signals, whereas m1A, m7G, and Ψ remain mainly at the level of basic mechanisms or cross-disease extrapolation [105,107]. Direct functional validation of m7G and Ψ in alveolar epithelial injury and aberrant repair is still lacking [107,108].

Finally, RNA modification-based therapeutic targeting faces translational barriers including low delivery efficiency, limited tissue specificity, potential systemic side effects, and insufficient biomarker-guided patient stratification [94,117]. For nucleic acid-based interventions, pulmonary delivery additionally requires optimization of formulation stability, airway deposition, mucus penetration, and intracellular release [93,96]. Overall, RNA modifications should currently be regarded as an emerging regulatory layer in pulmonary fibrosis translational research rather than a mature clinical therapeutic target axis [115,116].

## 11. Current Limitations and Future Perspectives: Four Key Scientific Gaps

Although RNA modifications have increasingly been investigated in alveolar epithelial injury, aberrant repair, and pulmonary fibrosis, the overall field is still transitioning from correlative descriptions toward mechanistic and functional validation [118,119].

Importantly, discrepancies among studies may partly arise from differences in disease models, cell composition, injury duration, and analytical platforms, highlighting the need to interpret RNA modification-associated findings within specific biological contexts [120].

Current major bottlenecks are mainly concentrated in insufficient cell-type specificity, incomplete mechanistic pathways, limited spatial and temporal resolution, and insufficient standardization of RNA modification quantification methods [121,122].

Moreover, evidence derived from human samples, animal models, and in vitro systems should be interpreted within their respective experimental contexts, because animal and in vitro models may not fully reproduce the chronic injury patterns, cellular composition, and epitranscriptomic heterogeneity of human pulmonary fibrosis [123].

### 11.1. Gap 1: Lack of AT2 Cell-Specific Causal Mechanistic Definition

Existing studies are mostly based on whole-lung tissues or mixed-cell systems, making it difficult to dissect the true regulatory roles of RNA modifications in AT2 cells and to determine whether findings obtained from different experimental systems, including human samples, animal models, and in vitro studies, are consistent [118,124].

Due to the dynamic changes in cellular composition during pulmonary fibrosis, transcriptional and epitranscriptomic signals from different cell types are extensively interwoven, leading to persistent uncertainty in cell-of-origin attribution in current datasets [119,124].

At present, a direct causal chain linking AT2 cell-specific RNA modification changes with defined target RNAs and measurable epithelial repair outcomes has not yet been established [125].

### 11.2. Gap 2: Insufficient Functional Evidence for Non-m6A RNA Modifications

Compared with m6A, the study of m5C, m1A, m7G, Ψ, and A-to-I RNA editing in pulmonary fibrosis remains in an early stage [126,127]. For example, base-resolution m1A mapping has revealed distinct m1A classes in nuclear- and mitochondrial-encoded transcripts, but these findings have not yet been translated into AT2 cell-specific fibrotic repair models [126].

Current studies are mainly based on expression changes and cross-disease correlative inferences, lacking systematic functional validation [127].

In particular, within the AT2 cell context, there is still a lack of site-level mechanistic resolution and genetically defined causal models [125,127].

Therefore, current evidence is insufficient to define the AT2-specific functional roles of non-m6A modifications or their direct contribution to epithelial repair failure [125,126].

### 11.3. Gap 3: Lack of Site-Level Functional and Causal Validation for RNA Modifications

Current studies mostly remain at the level of global modification changes or transcript enrichment analyses, lacking single-nucleotide resolution mechanistic interpretation [121,122].

Existing technologies such as MeRIP-seq provide limited resolution, and CLIP-based approaches have insufficient tissue specificity, which restricts precise identification of functional modification sites; antibody-free strategies such as DART-seq and standardized LC–MS/MS workflows partially address these limitations by enabling transcriptome-wide m6A detection and reproducible modification quantification [122,123].

Therefore, it remains unclear which RNA modification sites are functionally relevant to AT2 repair responses and fibrosis-associated phenotypes [125].

### 11.4. Gap 4: Lack of Single-Cell and Spatial Epitranscriptomic Atlas

Although single-cell RNA sequencing and spatial transcriptomics have been widely applied in pulmonary fibrosis research, single-cell and spatial resolution studies of RNA modifications remain limited [120,124].

Key unresolved gaps include the dynamic RNA modification trajectories of AT2 cells, the modification features of KRT8^+^ transitional states, and spatial gradients of RNA modifications within fibrotic niches, which will require integration of spatially resolved disease atlases with emerging single-cell epitranscriptomic technologies [124,126]. Methodological advances in quantitative RNA modification mapping, including mass spectrometry, antibody-based and non-antibody-based sequencing, nanopore direct RNA sequencing, and single-cell or single-molecule imaging, may provide complementary tools for resolving modification dynamics and cellular heterogeneity [121,122].

Therefore, an integrated epitranscriptomic atlas incorporating spatial, temporal, and modification-level dimensions has not yet been established [123,125].

### 11.5. Overall Perspective

Future RNA modification research should focus on four major directions:

(1) Elucidating AT2 cell-specific mechanisms and the context-dependent functions of KRT8^+^ transitional epithelial populations
(2) Establishing site-level functional validation systems, particularly for non-m6A modifications
(3) Integrating single-cell and spatial multi-omics analyses across human samples and disease models
(4) Developing cell-type-specific delivery strategies and evaluating therapeutic safety, efficacy, and clinical relevance

The ultimate goal is to move the field from correlative description toward cell-type-specific, site-resolved, and functionally validated evidence, thereby improving the biological interpretation and translational relevance of RNA modification research in aberrant alveolar epithelial repair [118,127].

## 12. Conclusions

Pulmonary fibrosis is increasingly understood as a multicellular remodeling process in which persistent alveolar epithelial injury and failed regeneration are closely associated with stromal activation and extracellular matrix deposition. AT2 cell dysfunction, impaired AT2-to-AT1 differentiation, and the persistence of KRT8^+^ transitional epithelial populations represent important features of aberrant repair. These populations should not be interpreted as established independent drivers of fibrosis, but rather as indicators of unresolved epithelial repair with potential context-dependent functional contributions. RNA modifications provide an additional post-transcriptional regulatory layer that may influence RNA fate, epithelial stress adaptation, senescence-associated responses, differentiation, and epithelial–mesenchymal communication. Among them, m6A currently has the strongest mechanistic support, whereas the roles of non-m6A modifications remain less well defined and require further functional validation.

Future research should move beyond correlative profiling toward cell-type-specific, site-resolved, and functionally validated evidence. Priority should be given to defining RNA modification events in AT2 cells, identifying their target transcripts and biological consequences, clarifying the context-dependent functions of KRT8^+^ transitional epithelial populations, and integrating human samples with lineage-resolved models and single-cell or spatial epitranscriptomic approaches. Translation will additionally require safe and cell-selective delivery systems, rigorous evaluation of therapeutic efficacy and off-target effects, and validation in clinically relevant pulmonary fibrosis models. These advances may clarify how epitranscriptomic regulation is associated with failed alveolar regeneration and whether selected RNA modification pathways may support epithelial repair-centered therapeutic strategies.

## Figures and Tables

**Table 1:** Major RNA modifications involved in alveolar epithelial injury and pulmonary fibrosis.

RNA Modification	Writers/Regulators	RNA-Level Function	Evidence in PF/AT2 Cells	**Re** **f.**
m6A	METTL3/METTL14/FTO/ALKBH5/YTHDF family	RNA stability, translation efficiency, and stress-response regulation	Regulates AT2 stress responses and epithelial repair failure	[31]
m5C	NSUN family/YBX1	RNA stability and nuclear export	Associated with epithelial stress and inflammatory activation in lung injury models	[32]
m1A	TRMT6/61A complex	Translation initiation and mitochondrial RNA regulation	Linked to metabolic stress adaptation in pulmonary epithelial cells	[33]
m7G	METTL1/WDR4	Translation efficiency and tRNA stability	Implicated in stress-induced translational reprogramming	[34]
Ψ	PUS family enzymes	RNA structural stability and translational fidelity	Contributes to RNA stress adaptation, with limited PF-specific evidence	[35]
A-to-I RNA editing	ADAR1/ADAR2	dsRNA sensing and innate immune regulation	Regulates immune homeostasis in lung injury and fibrosis models	[36]

Note: This table summarizes the major RNA modification types, their representative regulators, functional roles at the RNA level, and current evidence supporting their involvement in alveolar epithelial injury and pulmonary fibrosis.

**Table 2:** Representative evidence of RNA modifications in pulmonary fibrosis-related epithelial repair.

RNA Modification	Molecular Regulators	Functional Role in Alveolar Epithelial Cells/PF Context	Evidence Context	Ref.
m6A	METTL3/METTL14/FTO/ALKBH5/YTHDF1–3	Regulates AT2 stress response, epithelial injury–repair imbalance, and transcript stability during fibrotic remodeling	Lung fibrosis + AT2 epithelial injury models	[31]
m5C	NSUN2/YBX1	Controls RNA stability and is associated with stress-associated inflammatory responses in lung injury models	Lung injury/fibrosis-associated transcriptomic studies	[32]
m1A	TRMT6/61A	Modulates translation initiation and mitochondrial RNA adaptation under metabolic stress	Cellular stress models (limited lung validation)	[33]
m7G	METTL1/WDR4	Regulates translation efficiency and stress-responsive protein synthesis	Stress-response/epithelial translational control models	[34]
Ψ	PUS enzymes	Enhances RNA structural stability and translational fidelity under stress conditions	General RNA stress biology (no direct PF validation)	[35]
A-to-I RNA editing	ADAR1/ADAR2	Modulates dsRNA sensing and innate immune activation in epithelial injury contexts	Lung injury/innate immunity models	[36]

Note: This table summarizes representative evidence linking RNA modifications to epithelial stress responses, aberrant repair, and pulmonary fibrosis-related regulatory contexts.

**Table 3:** Experimental models and multi-omics approaches for studying RNA modification-associated AT2 injury and aberrant repair in pulmonary fibrosis.

**Category**	**Key Tools/Systems**	**Advantages**	**Limitations**	**Re** **f.**
AT2 cell-specific model systems	AT2-specific Cre-loxP models, primary AT2 culture, alveolar organoids, and iPSC-derived alveolar epithelium	Enables cell-autonomous interrogation of AT2 injury and repair dynamics and recapitulates epithelial state transitions	Limited physiological complexity in vitro and incomplete microenvironmental reconstruction	[97]
In vivo fibrosis models	Bleomycin-induced fibrosis and injury–repair models	Recapitulates multicellular fibrosis progression and epithelial injury–repair coupling	Lacks AT2-specific resolution and may introduce systemic injury bias	[97]
Single-cell transcriptomics and lineage tracing	scRNA-seq, RNA velocity, and lineage-tracing systems	Resolves epithelial heterogeneity, AT2-to-AT1 trajectory dynamics, and transitional states such as KRT8^+^ cells	Provides static or inferred trajectories and has limited temporal resolution without longitudinal validation	[52]
Spatial transcriptomics	Spatial RNA mapping platforms	Defines spatial coupling between aberrant epithelial states and fibrotic niches	Limited resolution for subcellular RNA dynamics and RNA modification sites	[98]
Epitranscriptomic profiling technologies	MeRIP-seq, LC–MS/MS, scDART-seq, and single-cell m6A sequencing	Enables global and single-cell-resolution mapping of RNA modification landscapes	Most platforms remain bulk-dependent or technically immature for non-m6A marks	[41]
Causal validation systems	Site-directed mutagenesis, AT2-specific genetic manipulation, rescue experiments, and time-resolved perturbation models	Supports validation of modification-dependent links among target RNAs, epithelial cell states, and fibrotic phenotypes	High experimental complexity and limited in vivo feasibility	[99]
Integrated multi-omics framework	scRNA-seq, epigenome, spatial, epitranscriptomic, and time-series integration	Reconstructs dynamic epithelial state trajectories and regulatory networks	Data integration complexity and dependence on computational inference	[94]

Note: This table summarizes representative model systems, multi-omics platforms, and validation strategies for investigating RNA modification-mediated regulation of AT2 cell injury and aberrant epithelial repair in pulmonary fibrosis.

## Data Availability

No new data were created or analyzed in this study. Data sharing is not applicable to this article.
